# *Schizophyllum commune* induced oxidative stress and immunosuppressive activity in *Spodoptera litura*

**DOI:** 10.1186/s12866-020-01831-6

**Published:** 2020-05-29

**Authors:** Mandeep Kaur, Pooja Chadha, Sanehdeep Kaur, Amarjeet Kaur, Rajvir Kaur

**Affiliations:** 1 Department of Zoology, Guru Nanak Dev University, Amritsar, Punjab India; 2 Department of Microbiology, Guru Nanak Dev University, Amritsar, Punjab India

**Keywords:** Basidiomycetes fungi, Polyphagous insects-pest, Antioxidant enzymes, Haemocytes

## Abstract

**Background:**

In the last few decades, considerable attention has been paid to fungal endophytes as biocontrol agents, however little is known about their mode of action. This study aimed to investigate the toxic effects of an endophytic fungus *Schizophyllum commune* by analyzing activities of antioxidant and detoxifying enzymes as well as morphology of haemocytes using *Spodoptera litura* as a model.

**Results:**

Ethyl acetate extract of *S. commune* was fed to the larvae of *S. litura* using the artificial diet having 276.54 μg/ml (LC_50_ of fungus) concentration for different time durations. Exposed groups revealed significant (*p* ≤ 0.05) increase in the activities of various enzymes viz. Catalase, Ascorbate peroxidase, Superoxide dismutase, Glutathione-S-Transferase. Furthermore, haemocytes showed various deformities like breakage in the cell membrane, cytoplasmic leakage and appearance of strumae in the treated larvae. A drastic reduction in the percentage of normal haemocytes was recorded in the treated groups with respect to control.

**Conclusion:**

The study provides important information regarding the oxidative stress causing and immunosuppressant potential of *S. commune* against *S. litura* and its considerable potential for incorporation in pest management programs.

## Background

Endophytes are ubiquitous in nature, forming associations with a diverse group of plant species without showing visible symptoms [1, 2]. Through a symbiotic relationship with their host plant, they enhance the plant’s tolerance to biotic and abiotic stresses. Various studies revealed that plants infected with endophytic fungi showed resistance to herbivory [3–5]. Several fungal species viz. *Cladosporium herbarum*, *Rhodotorula rubra*, *Alternaria alternata*, *Epicoccum nigrum*, *Penicillium* sp., *Fusarium graminearum, Cryptococcus* spp., have been reported to protect plants from herbivores [6, 7]. Some fungal endophytes act as insect pathogenic agents and have been reported to infect different insect species including lepidopterous larvae, aphids, thrips and many other cosmopolitan insects, which have a great concern in agriculture worldwide. They infect specific hosts, attributing little or no threat to non-target organisms or beneficial insects [8]. The anti-insect properties of endophytes have been documented by various researchers [9–11].

Many insect pathogenic fungi such as *Beauveria bassiana, Clonostachys rosea, Isaria farinosa*, *Fusarium oxysporum, Hypocrea lixii, Gibberella moniliformis*, and *Trichoderma asperellum* have been isolated as naturally occurring endophytes from asymptomatic plant tissues [12–17]. The insecticidal activity of fungi can be attributed to different secondary metabolites produced by them. Mycotoxins viz. aflatoxins, fumonisins, ochratoxins, zearalenone, have great importance in agriculture for pest management [18]. Various fungal secondary metabolites like avermectins, pantherine, destruxins, ibotenic acid, and tricholomic acid were found to be highly active against insects [19].

Although a lot of research has been done on the role of fungal endophytes as insect pathogenic agents but many of them failed to address the mode of action [20–22]. In order to discover the insecticidal potential, the effect on antioxidant and detoxifying enzymes in insects should be evaluated. In healthy animals, a balance between the production and elimination of reactive oxygen species (ROS) occur but an imbalance between the production and detoxification of ROS by the biological system results in oxidative stress [23]. Insects have a complex enzymatic and non-enzymatic defense system to encounter oxidative stress. The system regulates the level of lipid peroxidation and prevents the damage to DNA, proteins and other cytotoxic effects [24]. The main antioxidant enzymes in insects are catalase (CAT), Ascorbate peroxidase (APOX) and superoxide dismutase (SOD) [24, 25]. SOD catalyzes the dismutation of superoxide radicals into oxygen and H_2_O_2_. CAT and APOX catalyze the dismutation of H_2_O_2_ into oxygen and water. Another detoxifying enzyme, glutathione-S-transferase (GST), eliminates hydroperoxides from the cells [26, 27]. Various xenobiotics incite the production of reactive oxygen species (ROS) and unbalance the antioxidant–pro-oxidant equilibrium, ultimately induce oxidative damage, cytotoxicity or immunotoxicity and an increase in insects’ mortality [28–31]. Such effects arise either from the pro-oxidant activity which can be observed as lipid peroxidation or altered antioxidant enzyme activity. So, these parameters are significant while estimating the stress caused by xenobiotics.

Insect haemocytes play an active role in providing immunity. They consist of a mixture of cells having different morphological and biological functions. The haemocytes are competent in discriminating stranger agents, mediates phagocytosis, encapsulation, cytotoxicity, wound repair and coagulation [32, 33]. So they play an important role in providing defense against parasites, pathogens and other foreign bodies entering hemocoel [34–37]. Several studies revealed the effect of biopesticides on haemocytes count but the morphological alterations in haemocytes have not yet been studied. As haemocytes play an important role in providing cellular immune defense, the negative impact on haemocytes also reflects immunosuppressant nature.

On the basis of the aforementioned discussion, the study was conducted to decipher the effect of ethyl acetate extract of *Schizophyllum commune* on the activity of antioxidant, detoxifying enzymes and morphology of haemocytes of *Spodoptera litura*, one of the major polyphagous pest.

## Results

**Fig. 1:**
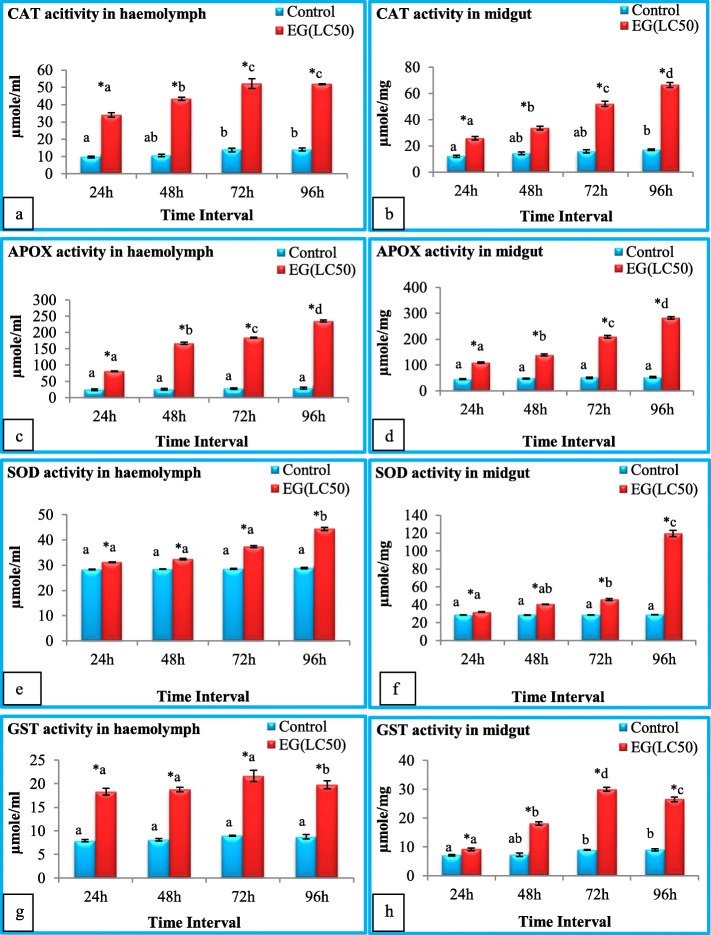
**a**-**h** Catalase (CAT), Ascorbate peroxidase (APOX), Superoxide dismutase (SOD), and Glutathione-S-Transferase (GST) activity in haemolymph and midgut of *S. litura after* treatment with ethyl acetate extract of *S. commune* at different time intervals. EG = Exposed group. Bars represent mean ± S.E. *Ascribes the significant difference between exposed group and control group (t-test, *p* ≤ 0.05). Different letters a, b, c, d are significantly different (Tukey’s test, *p* ≤ 0.05) and signify the effect of duration. The enzyme activity was expressed as μmole/ml (haemolymph) and μmole/mg (midgut) weight

**Fig. 2:**
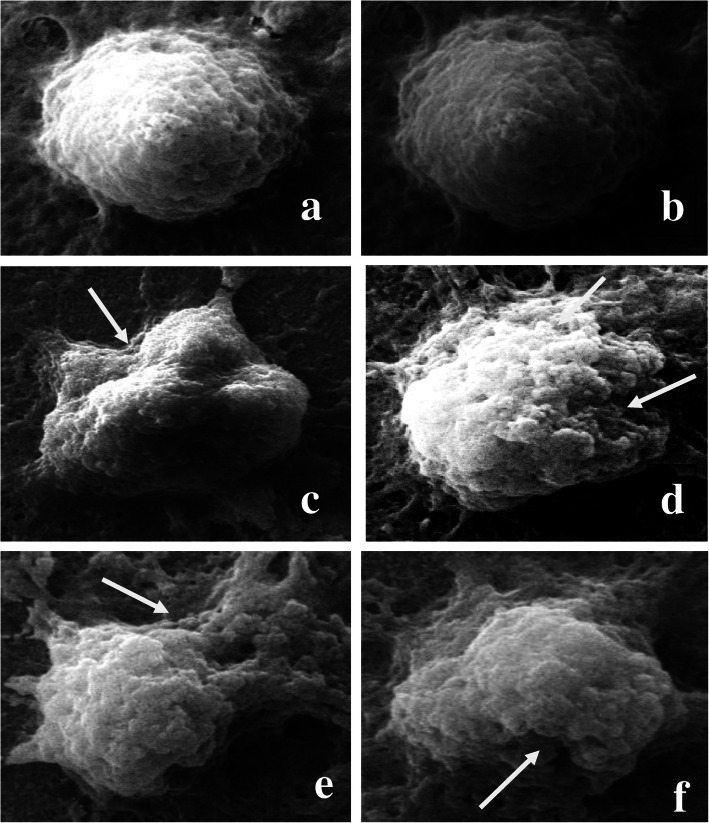
Microphotographs showing haemocytes (Plasmatocytes) (**a**-**b**). Normal haemocyte; (**c**-**f**). Various deformities observed in haemocytes after treatment with ethyl acetate extract of *S. commune*; **c**. Cell membrane shrinkage of haemocyte; **d**-**f**. Breakage in membrane and cytoplasmic leakage

**Fig. 3:**
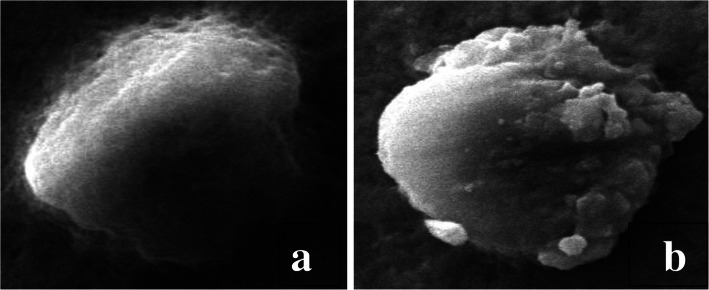
Microphotographs showing haemocytes (Granulocytes) (**a**). Normal haemocyte; (**b**). Strumae and surface abnormalities in haemocytes after treatment with ethyl acetate extract of *S. commune*

**Fig. 4:**
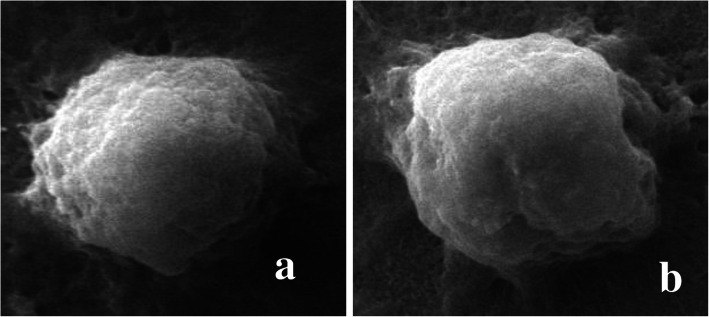
Microphotographs showing haemocytes (prohaemocyte) (**a**). Normal haemocytes (**b**). Cell membrane shrinkage in haemocyte after treatment with ethyl acetate extract of *S. commune*

**Fig. 5:**
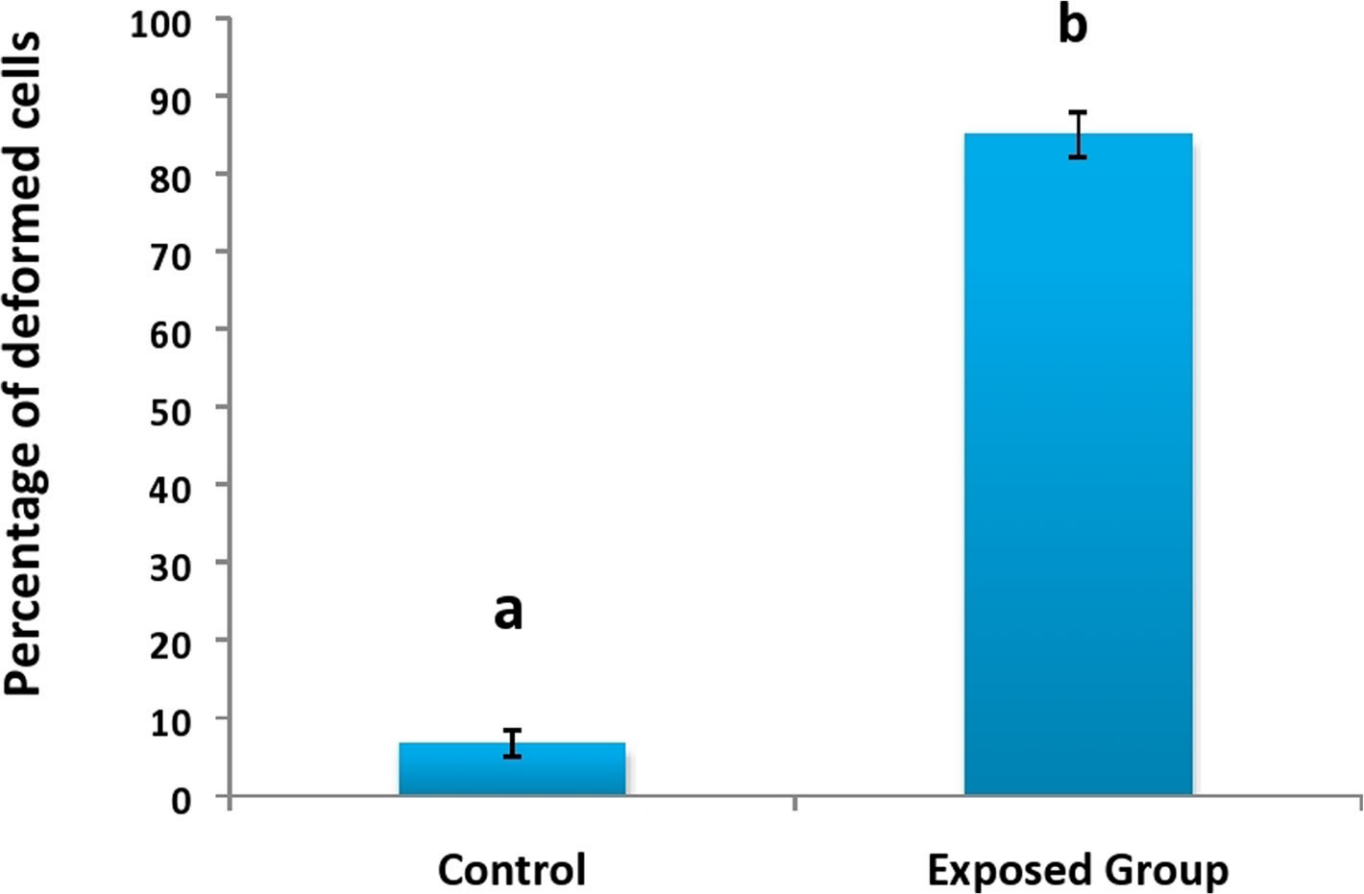
The percentage of cells showing various deformities

The results of the present study have shown in Fig. 1a-h and Figs 2, 3, 4 and 5.

Fig. 1a, b reveals a significant increase (t-test, *p* ≤ 0.05) in catalase (CAT) activity in both haemolymph and midgut tissue of treated larvae, as compared to control. The maximum hike was observed at 96 h, where values are 3.68 and 3.87 fold higher in haemolymph (Fig. 1a) and midgut (Fig. 1b) of treated larvae as compared to control, respectively. ANOVA followed by Tukey’s test revealed the significant (p ≤ 0.05) effect of duration of exposure for both tissues.

Change in Ascorbate peroxidase (APOX) activity in both tissues due to ingestion of ethyl acetate extract of *S. commune* is given in Fig. 1c, d. After 24 h of exposure, the value of APOX increased 3.37 fold in haemolymph and 2.43 fold in midgut tissue as compared to control. Furthermore, an increase in the activity was observed with increase in the duration of exposure. The value was found to be increased by 8.01 times in haemolymph (Fig. 1c) and 5.32 times in midgut (Fig. 1d) of 96 h exposure time group as compared to control. Significant (Tukey’s test, *p* ≤ 0.05) effect of duration was observed in both haemolymph and midgut.

Similar effects were observed on the activities of Superoxide dismutase (SOD) (Fig. 1e, f) and Glutathione-S-Transferase (GST) (Fig. 1g, h). The values of SOD and GST were increased significantly (t-test, *p* ≤ 0.05) in all exposure time groups as compared to control, in both the tissues investigated. The SOD activity was maximum at 96 h exposure group where 1.53 fold (haemolymph) (Fig. 1e) and 4.14 fold (midgut) (Fig. 1f) increase was observed as compared to control groups. The GST activity increased up to 72 h and then a slight decrease was observed in 96 h exposure group in both tissues (Fig. 1g, h). The effect of duration of exposure was found to be significant (Tukey’s test, *p* ≤ 0.05) for SOD activity in both tissues and for GST activity in midgut tissue. GST activity increased non-significantly in the haemolymph of treated groups.

SEM studies revealed various morphological deformities in haemocytes of 96 h treated larvae of *S. litura.* As compared to normal plasmatocytes, treated ones showed cell membrane shrinkage, breakage in the membrane and cytoplasmic leakage (Fig. 2). Similarly, granulocytes also showed strume and surface abnormalities in the treated group (Fig. 3).

Cell membrane shrinkage was also observed in prohaemocytes of treated larvae (Fig. 4). Overall the morphology of haemocytes was found to be highly disrupted after treatment with ethyl acetate extract of *S. commune* for 96 h which may ultimately lead to the immunosuppressive effect.

Relative to the control, the percentage of haemocytes showing various deformities were significantly increased in treated larvae because of the toxic effects of the fungal extract. After 96 h of feeding, the percentage of cells showing various deformities was 85.00% as compared to 6.66% in control (Fig. 5).

## Discussion

In the last few decades, endophytic fungi have attracted considerable attention as biocontrol agents in sustainable agriculture. They are known to have several advantages as compared to synthetic pesticides [14]. To date, several studies have demonstrated the fungal endophytes as insect pathogens but many of them failed to address the mode of action [20–22].

Insects have evolved multiple defense mechanisms including antioxidant and cellular immune defense, to respond to pathogens [24, 25, 32, 33]. Antioxidant defense system in insects consists of a network of protective enzymes such as Catalase (CAT), Ascorbate peroxidase (APOX), Superoxide dismutase (SOD) and Glutathione-S-Transferase (GST) which work coordinately to maintain the state of dynamic equilibrium in organisms, keeping reactive oxygen species (ROS) level low to prevent the oxidative stress induced cellular damage [24]. SOD catalyzes the dismutation of superoxide anion to hydrogen peroxide, which is subsequently detoxified to oxygen and water by catalase, ascorbate peroxidase or GST. Previous studies have demonstrated that, after fungal infection, protection systems of insects are activated to ward off infection and to maintain the normal physiological activities [38–42]. In our study, a significant increase in CAT, APOX and SOD activities were noticed in larvae of *Spodoptera litura* after treatment with ethyl acetate extract of *Schizophyllum commune*. Similarly, Karthi et al. [43] reported the increase in superoxide dismutase (SOD), catalase (CAT), peroxidases (POX) activity under the influence of entomopathogenic fungus *Aspergillus flavus* in *S. litura*. In our research, results showed that, at 96 h, the activities of CAT, APOX and SOD have reached their maxima. This might be due to the activation of protective enzymes after stimulation by *S. commune* treatment to defend the body against oxidative damage. The three enzymes work synergistically, keeping the ROS level low and prohibiting oxidative damage. The overall activity trends were consistent with previous research [38, 44–51].

GST as a detoxifying enzyme effectively metabolizes the exogenous toxic compounds and plays a crucial role in providing defense as well as maintaining the normal physiological activities in the body [52–54]. The enzyme catalyzes the harmful compounds with glutathione and assists their discharge from the body in a non-enzymatic fashion [55]. In this research, an upsurge in GST activity was noticed when larvae of *S. litura* were fed with diet having ethyl acetate extract of *S. commune*. At 72 h, GST activity reached its maximum level. However, at 96 h, a slight decrease was observed. The results were according to an earlier report which indicated the elevated activity of GST in response to entomopathogenic fungus *Beauveria bassiana* in *Andrallus spinidens* (Fabricius) [56]. Ding et al. [38] and Jia et al. [57] observed a similar trend in GST activity after exposure to fungus *B. bassiana* and *Metarhizium anisopliae* in *Xylotrechus rusticus* (Linnaeus) and *Locusta migratoria* (Linnaeus), respectively.

Cellular immune defense in insects is because of different haemocytes which play a vital role in providing immunity to insects against pathogens. There are different classes of haemocytes which have been morphologically and functionally characterized in various insects [33, 58–64]. The most common types of haemocytes reported in the literature are prohemocytes, granulocytes, plasmatocytes, and oenocytoids. Their multifunctional roles are phagocytosis, encapsulation, cell agglutination, detoxification, etc. [58–61]. Change in number and configuration of haemocytes ultimately affect the immunity and health of insects [65, 66].

Scanning electron microscopic observations in the present study revealed various morphological deformities within haemocytes of *S. litura* larvae, exposed to ethyl acetate extract of *S. commune*. The percentage of normal haemocytes was found to be highly reduced in the treated groups. As compared to control, various cellular deformities observed in treated ones are cell perforations, rupturing of haemocytes with cytoplasmic leakage and irregular variation on the surface of haemocytes. Many of them showed resemblance with changes observed in haemocytes of *Bombyx mori* (Linnaeus) after exposure to destruxin A [67]. There are very fewer reports available in literature revealing the abnormalities of insect haemocytes using SEM. This is the first finding reporting the alterations in different types of haemocytes in *S. litura* under SEM. The technique has been used to observe and characterize the haemocytes of different insects in various reports [68, 69] and also to observe the spores accumulation in the body of the insect after fungal infection [70–73]. However, similar types of morphological changes as observed in the present finding were demonstrated by other workers due to entomopathogenic fungi and insecticides under light microscopy [74–77].

## Conclusion

All these results suggest that *S. commune* infects *S. litura* by directly acting on the antioxidant and cellular immune defense, resulting in oxidative stress and decreased immune function. Overall, the study provides important information about the oxidative stress causing and the immunosuppressant potential of *S. commune* against *S. litura* and its considerable potential for incorporation in pest management programs.

## Methods

### Rearing of *Spodoptera litura*

*Spodoptera litura* (Lepidoptera) eggs were obtained from the cauliflower fields around Amritsar (India). After hatching of eggs, larvae were fed on castor leaf. Subsequent generations of the culture were maintained in the laboratory at 25 ± 2 °C temperature, 65 ± 5% relative humidity and 12:12 (D: L) photoperiod [78].

### Fungal culture isolation, production and identification

Endophytic fungus was isolated from leaves of *Aloe vera* collected from Amritsar (India). The leaves were thoroughly washed with distilled water, followed by sterilization with 70% ethanol (2 min), 5% sodium hypochlorite solution (5 min) and finally rinsed with sterile distilled water. Sterilized samples were cut into small pieces, and they were placed on water agar plates having ampicillin (200 mg/ml) as an antibacterial agent and incubated at 30 °C. After the emergence of hyphae, the hyphae tips were picked and cultured on PDA (potato dextrose agar) plates. Then the culture was purified and maintained on PDA for further studies [79].

The production was carried out in 50 ml malt extract (malt extract = 20 g/l, dextrose = 20 g/l, peptone = 1 g/l, pH = 5.5) broth in 250 ml Erlenmeyer flask by inoculating one plug (1 cm square) taken from the periphery of an actively growing culture. The flasks were incubated at 30 °C and 250 rpm for 10 days. After 10 days extraction was carried out twice using ethyl acetate at 120 rpm and 40 °C. The extracts were concentrated by using rotavapor and dissolved in 1 ml DMSO and stored at 4 °C. The fungus was identified as *Schizophyllum commune* on morphological (Fig S1: Morphology of *S. commune* showing hyphae and clamp connection, characteristics of basidiomycetes under SEM) and molecular basis as indicated in our previous study [80] by using ITS1 and ITS4 primers to amplify ITS1–5.8S- rDNA- ITS2 region. Amplified ITS region was Purified and sequenced at first base sequencing (Malaysia). The sequence similarity was matched with other available databases retrieved from NCBI using BLAST [81]. The sequence was deposited into GeneBank under accession number: MF680077.

### Toxicity test

On the basis of bioassay studies, the LC_50_ value of ethyl acetate extract of *S. commune* was found to be 276.542 μg/ml [80]. This concentration is selected for, to analyze its effect on antioxidant and detoxification enzymes and to decipher various morphological changes in haemocytes.

### Antioxidant enzyme activities

To evaluate the effect of fungal extracts on antioxidant enzymes, the third instar larvae (12 days old) were fed with fungal extracts supplemented diet having a concentration 276.54 μg/ml. The enzyme activities [Superoxide dismutase (SOD), catalase (CAT), Ascorbate peroxidase (APOX) and Glutathione-S-Transferase (GST)] were analyzed in haemolymph and midgut of third instar (12 days) larvae.

Larvae were divided into two groups, treatment and control. The treatment group was exposed with LC_50_ of fungus at controlled temperature 25 ± 2 °C and relative humidity 65 ± 5%. The second group was treated with control diet (0.5% DMSO) at the same conditions of temperature and relative humidity. The effect of fungal extract has been recorded after different time intervals (24 h, 48 h, 72 h and 96 h) in enzyme activities. The experiment was replicated three times. For each treatment and control, there are 10 larvae per replication were taken.

### Tissue collection

Haemolymph was collected by cutting prolegs with microscissor from 10 different larvae fed with the same concentration and then it was pooled. Pooled haemolymph (10%) was mixed with PBS (Phosphate Buffer Saline pH 7.0) containing 0.01% phenylthiourea and centrifuged for 20 min at 10000 g, 4 °C and supernatant obtained was used for enzyme activities studies. Similarly, midgut tissue was also taken after dissection with microscissor from 10 different larvae fed with the same concentration and homogenate (10% w/v) was prepared by homogenizing larval midguts (100 mg in 1 ml) in PBS. Afterwards, the homogenate was centrifuge in PBS for 20 min at 10000 g, 4 °C and supernatant obtained was used for enzyme activities studies.

The extraction procedure was the same for all enzymes.

### Catalase (CAT) activity

Enzyme activity was estimated according to the methodology given by Aebi [82] with slight modifications. 0.1 ml of supernatant was added into 2.9 ml of H_2_O_2_ in a cuvette. The decrease in absorbance was read at 240 nm for 5 min at 1 min interval (25 °C). The enzyme activity was expressed as μM/ml (haemolymph) and μM/mg (midgut) weight.

### Ascorbate peroxidase (APOX) activity

The enzyme activity was calculated according to the methodology introduced by Asada [83] with slight modifications. 0.1 ml of sample, 0.6 ml extraction buffer (50 mM potassium phosphate buffer pH 7.0) and 0.125 ml of 0.3%H_2_O_2_ were taken in a cuvette. The decrease in absorbance was recorded at 290 nm for 5 min at 30s interval (25 °C). The enzyme activity was expressed as μM/ml (haemolymph) and μM/mg (midgut) weight.

### Superoxide dismutase (SOD) activity

The enzyme activity was calculated according to the methodology followed by Kono [84] with slight modifications. 0.05 ml sample, 1.5 ml extraction buffer (50 mM sodium carbonate buffer pH 10.0), 0.5 ml of 96 μM NBT (Nitroblue tetrazolium), 0.1 ml TritonX-100, 0.1 ml of 20 mM hydroxylamine hydrochloride were taken in cuvette and increase in absorbance was recorded at 540 nm. The enzyme activity was expressed as μM/ml (haemolymph) and μM/mg (midgut) weight.

### Glutathione- S-transferase (GST) activity

GST activity was estimated using the method of Habig et al. [85] with minor modifications. 50 μl of 10 mM CDNB (1- chloro-2, 4-dinitrobenzene), 100 μl GSH (Reduced glutathione), 50 μl of sample and 0.2 ml of 0.1 M sodium phosphate buffer containing PTU (phenylthiourea) were incubated at 25 °C and absorbance change was recorded at 340 nm for 5 min at 1 min interval. The enzyme activity was expressed as μM/ml (haemolymph) and μM/mg (midgut) weight.

### Effect on the morphology of haemocytes

To study the morphological alterations in haemocytes, the methodology of Wang et al. [86] with slight modifications was followed. The haemolymph of insects exposed for 96 h was bled on termanox discs after cutting prolegs of larvae. It was allowed to dry and fixed with 2.5% glutaraldehyde in 0.1 M cacodylate buffer (pH 7.2) for 2 h. After this, sequential dehydration was done by using a graded series of 25% ethanol followed by 50, 70, 90% and at the end with absolute (100%) alcohol. Then discs were placed in a dry chamber for proper drying. At the end silver coating was done by mounting samples on aluminium stubs and haemocytes were observed under SEM at a magnification of 10.00KX operated at 10KV. The percentage of cells showing various deformities were also calculated in the treatment and control group after 96 h exposure to *S. commune* ethyl acetate extract.

### Statistical analysis

To study the effect of duration one way analysis of variance (ANOVA) with Tukey’s test was performed and to study the effect of treatment student’s t-test was applied.

## Supplementary information

**Additional file 1: Figure S1.** Morphology of *S. commune* showing hyphae and clamp connection (characterstics of basidiomycetes) under SEM.

## Data Availability

All data generated or analyzed during this study are included in this article and its additional files.

## Abbreviations

CAT: Catalase
APOX: Ascorbate peroxidase
SOD: Superoxide dismutase
GST: Glutathione- S-Transferase
