# Supplementary material for: Schizophyllum commune induced oxidative stress and immunosuppressive activity in Spodoptera litura
Source: BMC Microbiol. 2020 May 29;20:139. doi: 10.1186/s12866-020-01831-6 (PMC7260734; doi:10.1186/s12866-020-01831-6)
Supplement: Supplementary file 1 — Additional file 1: Figure S1. Morphology of S. commune showing hyphae and clamp connection (characterstics of basidiomycetes) under SEM. [file 12866_2020_1831_MOESM1_ESM.docx]

**Additional information:**





**Fig S1:** Morphology of *S. commune* showing hyphae and clamp connection (characterstics of basidiomycetes) under SEM.
